# Selection of long oligonucleotides for gene expression microarrays using weighted rank-sum strategy

**DOI:** 10.1186/1471-2105-8-350

**Published:** 2007-09-19

**Authors:** Guangan Hu, Manuel Llinás, Jingguang Li, Peter Rainer Preiser, Zbynek Bozdech

**Affiliations:** 1 School of Biological Sciences, Nanyang Technological University, No. 60 Nanyang Drive, 637551, Singapore; 2 Department of Molecular Biology, Lewis-Sigler Institute for Integrative Genomics, Princeton University 246 Carl Icahn Laboratory, Princeton NJ 08544, USA; 3 Department of Pathology & Laboratory Medicine, Tan Tock Seng Hospital, 11 Jalan Tan Tock Seng, 308433, Singapore

## Abstract

**Background:**

The design of long oligonucleotides for spotted DNA microarrays requires detailed attention to ensure their optimal performance in the hybridization process. The main challenge is to select an optimal oligonucleotide element that represents each genetic locus/gene in the genome and is unique, devoid of internal structures and repetitive sequences and its Tm is uniform with all other elements on the microarray. Currently, all of the publicly available programs for DNA long oligonucleotide microarray selection utilize various combinations of cutoffs in which each parameter (uniqueness, Tm, and secondary structure) is evaluated and filtered individually. The use of the cutoffs can, however, lead to information loss and to selection of suboptimal oligonucleotides, especially for genomes with extreme distribution of the GC content, a large proportion of repetitive sequences or the presence of large gene families with highly homologous members.

**Results:**

Here we present the program OligoRankPick which is using a weighted rank-based strategy to select microarray oligonucleotide elements via an integer weighted linear function. This approach optimizes the selection criteria (weight score) for each gene individually, accommodating variable properties of the DNA sequence along the genome. The designed algorithm was tested using three microbial genomes *Escherichia coli*, *Saccharomyces cerevisiae *and the human malaria parasite species *Plasmodium falciparum*. In comparison to other published algorithms OligoRankPick provides significant improvements in oligonucleotide design for all three genomes with the most significant improvements observed in the microarray design for *P. falciparum *whose genome is characterized by large fluctuations of GC content, and abundant gene duplications.

**Conclusion:**

OligoRankPick is an efficient tool for the design of long oligonucleotide DNA microarrays which does not rely on direct oligonucleotide exclusion by parameter cutoffs but instead optimizes all parameters in context of each other. The weighted rank-sum strategy utilized by this algorithm provides high flexibility of oligonucleotide selection which accommodates extreme variability of DNA sequence properties along genomes of many organisms.

## Background

DNA microarray is one of the most powerful and versatile tools for post-genomic research [1]. After the initial success with cDNA and PCR product-based microarrays, application of long oligonucleotides became widely used in "spotted" DNA microarray technology in the last five years [2-5]. From the beginning it became clear that the design of the oligonucleotide probes requires special attention. Under a single stringency condition, hybridization specificity and efficiency of all oligonucleotides must be globally maximized across the entire array. Thus for the selection of the optimal oligonucleotide candidates, four major parameters are being evaluated: (i) uniqueness which analyzes other possible cross-hybridization targets in the genome; (ii) sequence complexity which evaluates the presence of short nucleotide repeats; (iii) melting temperature (Tm) or GC content which ensures a uniform hybridization efficiency across the microarray; and (iv) level of internal secondary structures which helps to avoid all possible self-binding interference with the specific target hybridization. In principle each of these properties can be calculated individually for every potential oligonucleotide candidate, however, the main challenge that remains is to derive a selection strategy that combines these parameters and selects the most optimal oligonucleotide representative for a given genetic locus/gene.

All currently available programs for long oligonucleotide microarray design utilize different parameters: the binding energy or BLAST-based score to alternative targets to evaluate uniqueness, the GC content or Tm to estimate hybridization stringency, the reverse Smith-Waterman score or free energy to evaluate levels of secondary structure and various types of complexity coefficients to evaluate the presence of short nucleotide repeats in each oligonucleotide element [5-11]. Typically these programs select one or more oligonucleotide representatives of a gene using various systems of cutoff-based filters. For example ArrayOligoSelector creates an intersection of oligonucleotides that pass parameter-based cutoffs for uniqueness, self-binding and sequence complexity. The intersection candidate list is then passed on to the GC filter and subsequently the final representative(s) are selected using a 3' proximity criteria [5]. The cutoff based algorithms provide a powerful approach to select DNA microarray oligonucleotide sets and were successfully used to design DNA microarrays for a large number of species [5,11-13]. The use of these algorithms is, however, not completely optimal for genomes with high abundance of repetitive sequences and large fluctuations of GC content. To accommodate such genomic sequences, the methods must relax the parameter filter adjustments. The wide "opening" of the cutoff filters can cause selection of suboptimal oligonucleotides for a significant number of genes, due to the fact that all oligonucleotides that pass a particular filter are treated as equal by the subsequent steps, disregarding their subtle diversity within the filtered interval of the parameter (unpublished observations).

To overcome these shortcomings new algorithms which incorporate optimization strategies of oligonucleotide parameters were developed including OligoDesign [14] and CommOligo [15]. OligoDesign was developed specifically for the design of the locked nucleic acid (LNA) microarray platform which takes advantage of the improved nucleic on-chip capture sensitivity of the LNA substitute mixmer oligonucleotides. Design of these specialized probes requires careful optimizations of the hybridization specificity and efficiency for each probe. For this purpose, OligoDesign uses an extensive fuzzification process derived from neural network approaches to ensure the optimal performance of this highly specialized microarray platform [14]. Similar to the fuzzy logic approach, CommOligo uses a piece-wise linear function to select optimal oligonucleotides via a user configurable iterative process [15]. Both of these methods represented a step in the right direction, recognizing the need for parallel optimization of all used parameters and elimination of cutoffs that cause information loss. At its presently available implementation, however, both OligoDesign and CommOligo utilize complex and computer-time consuming processes that render them unsuitable for high throughput applications. Nevertheless both methods have been useful for design of focused "miniarrays" which typically contain smaller numbers of genes *e.g*. 120 stress response and toxicological markers from *Caenorhabditis elegans *[14] or microarrays for relatively small genomes such as *Methanoccocus maripaludis *with 1759 genes [15].

Here we present a novel program named OligoRankPick that is inspired by the aforementioned parameter optimization approaches and it is suitable for the design of gene specific long oligonucleotide probes for genomes of all sizes. The final decision making process is based on a weighted rank-sum strategy which significantly streamlines the entire computation process. This complete eliminates all cutoff based filters, thereby significantly improves the quality of the resulting microarray oligonucleotide design. Moreover, the weighted rank-sum approach enables us to implement an integer weighted linear function to automatically optimize the oligonucleotide parameters for each gene individually. Finally to demonstrate the utility of OligoRankPick we design, assemble and verify a new version of *P. falciparum *microarray which comprises of 10166 oligonucleotides representing 5363 genes.

## Results

### Algorithm overview

**Figure 1:**
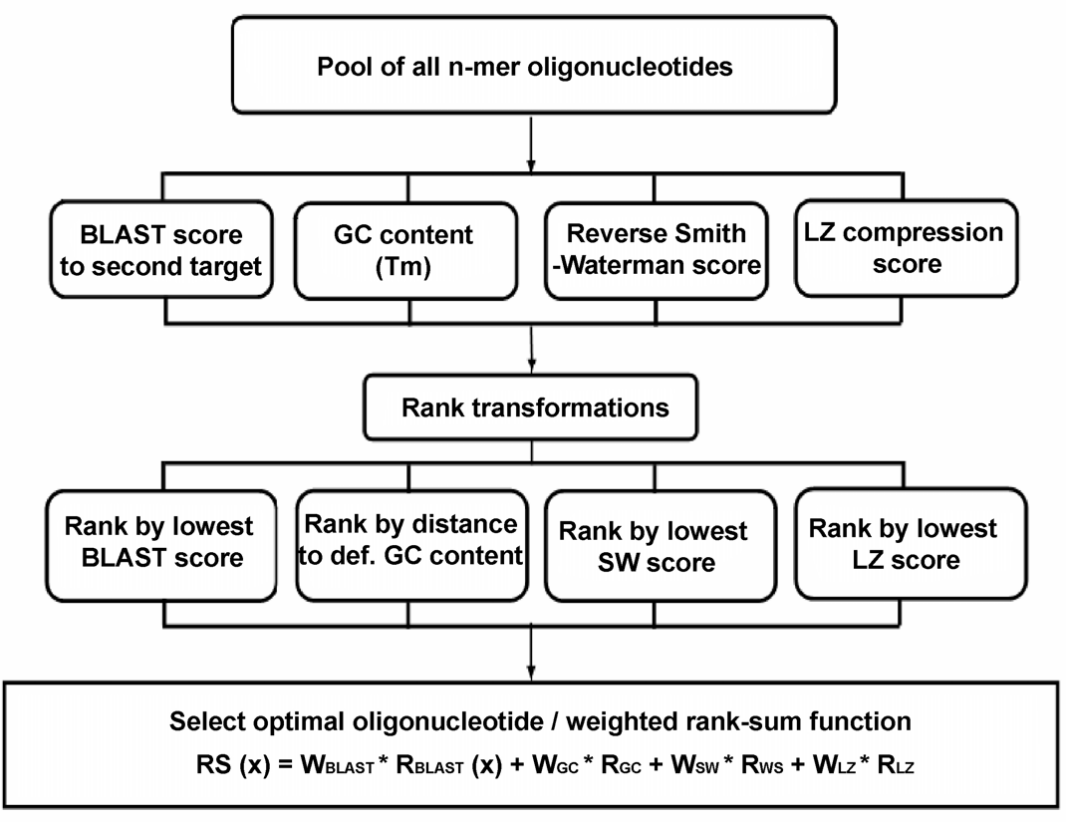
The flowchart of OligoRankPick. All possible oligonucleotides were extracted form the input sequence and stored. Subsequently four parameters of all possible oligonucleotides were calculated including the BLAST score to a second genomic target (uniqueness), the GC content (Tm), the Reverse Smith-Waterman score (self-binding) and the LZ compression score (sequence complexity). In the rank transformation step, the oligonucleotides are ranked based on each parameter and ordinal rank number is given to all oligonucleotides in each parameter rank independently. Finally weighted rank-sum (RS_(x)_) is calculated for all oligonucleotides with uniqueness weights (W_BLAST_), GC content weights (W_GC_) self-binding weights (W_SW_), and sequence complexity weights (W_LZ_) and R_BLAST_, R_GC_, R_SR _and R_LZ _representing the ranks corresponding to each parameter ranking. Multiple RS_(x) _are determined by the gene specific optimization using multiple weight sets (not indicated) and the lowest value is finally considered. The optimal candidate is selected based on the lowest RS_(x) _amongst all oligonucleotides in the locus.

Figure 1 summarizes the global overview of the OligoRankPick algorithm. Essentially, all possible oligonucleotide windows from a gene/locus are extracted and scored by the four parameter measurements, uniqueness (BLAST score to second target), GC content (GC content, Tm), self-binding (Reverse Smith-Waterman, SW) and sequence complexity (Lempel-Ziv compression score) (figure 1). Subsequently, each score is transformed into a rank and a weighted rank-sum is calculated for each oligonucleotide using the weighted optimization strategy (see below). The final oligonucleotide is selected based on the smallest rank-sum value.

### Rank transformation

Several previous methods have been introduced to normalize oligonucleotide parameter scores including a piece-wise, and sigmoid function [14,15]. In our approach the transformations of score values calculated for each parameter into rank numbers allows us to uniformly assesses and adjust the contribution of different parameters for the optimal oligonucleotide selection. For all coding sequences of the test genome (*P. falciparum*) there is a complete agreement between the overall profiles of the scores and the converted ranks in each parameter (Spearman rank order test, P≅0, see Additional file 1 figure S1). This indicates that the rank transformation does not affect the oligonucleotide status in the oligonucleotide set, and that the rank transformation has an equal power of parameter comparisons to the original scores.

### Optimizations of weight sets

The challenge of deriving weight values that will select an optimal oligonucleotide in the rank-sum strategy is two-fold: (i) the weight coefficients should correspond to the relative contribution of each parameter to the oligonucleotide performance during the microarray hybridization (formula 1); (ii) the weight set optimization should provide sufficient flexibility to accommodate the variable nature of the primary structure along the genome. For this purpose we aimed to develop a strategy that optimizes the weight set for each gene individually by considering broader intervals of weight coefficients rather then a single target value (formula 2).

To derive and subsequently evaluate this strategy, we used the *P. falciparum *genome which is characterized by large fluctuations of GC content, and an abundance of repetitive sequences and large highly homologous gene families [16]. In the first step we calculated the top oligonucleotide for all *Plasmodium *genes using 162 different weight sets. These sets originate from all combinations of four weight intervals: w_BLAST_=[1,2,3,4,5,6,7,8,9], w_GC_=[1,2,3,4,5,6,7,8,9], w_SW_=[1,2], w_LZ_=[1]. The broader intervals for uniqueness and CG content weight coefficients are intended to allow higher impact of these parameters on the final oligonucleotide selection. These adjustments are based on our previous observations, indicating that variations in GC content and uniqueness have a greater effect on specificity and efficiency of microarray hybridization compared to the other parameters [2,17] and Bozdech *et al *unpublished data). The implementation of weight interval optimization strategy (formula 2) then facilitates the gene specific optimizations of the oligonucleotide selection with respect to the sequence properties of a particular gene.

**Figure 2:**
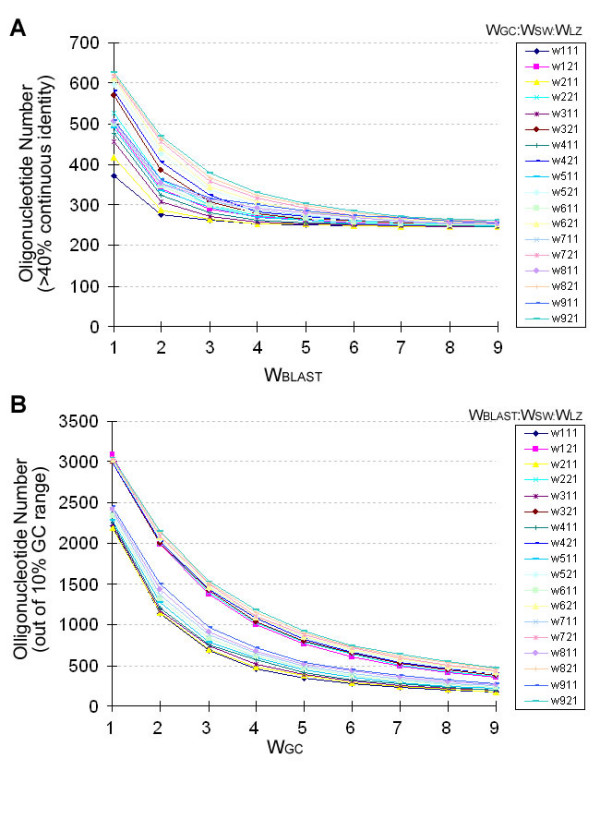
Number of oligonucleotides outside of the quality control criteria ploted along variable weight sets. The theoretical microarray oligonucleotide sets for the *P. falciparum *genome were designed using 162 weight sets assembled from intervals of uniqueness weights (W_BLAST_), GC content weights (W_GC_) self-binding weights (W_SW_), and sequence complexity weights (W_LZ_). In these number of oligonucleotides rejected based on the 40%-uniqueness rule (> 40% continuous sequence identity to a second target) was determined and plotted for each weight set. Each curve represents a profile of rejected oligonucleotide numbers with increasing uniqueness weights while the other weights are kept constant **(A)**. Similar to the uniqueness weight profiles number of oligonucleotides rejected using the GC content range rule (GC content outside of the 31.4% ± 5%) were plotted with the increased CG content weights **(B)**.

Previous studies suggest that 40% identity of continuous match is an upper limit for possible microarray cross-hybridization signal for oligonucleotides between 50 and 70 nucleotides (nt) [2,5,17]. For the GC content a 5% deviation (10% range) will result in a fluctuation in Tm of 4°C which provides a theoretical upper limit for microarray hybridization stringency (default setting in most programs). We utilized these two rules (< 40% of continuous match to a second target and 5% of GC content deviation from the target (31.4%)) as criteria to evaluate the quality of oligonucleotide selection (figure 2). It is important to emphasize that these criteria are not implemented as cutoff values in the OligoRankPick algorithm but as quality monitoring criteria to evaluate the selection process utilizing varying weight sets. First 162 oligonucleotide sets of the *P. falciparum *genome were generated using all combinations of the initial weight values. Then the number of oligonucleotides that passed the both rules was calculated (figure 2). In the uniqueness weight simulations the number of oligonucleotides outside of the limit criteria gradually plateaus for the uniqueness weights (w_BLAST_) greater than 6. For w_BLAST _values greater then 9 there were essentially no further oligonucleotide attritions regardless of the other weight values (figure 2A). By manual inspection we observed that the non-unique oligonucleotides (for w_BLAST _> 6) originate mostly from duplicated genes for which no unique oligonucleotides could be selected. The GC content modeling exhibits essentially identical tendencies with the vast majority of oligonucleotides that were found outside of the GC range originating from genes with an extremely high AT content (figure 2B). Taken together both GC content and uniqueness weight values bellow 6 do not generate enough power to maximize these parameters while values greater then 9 do not improve the numbers of acceptable oligonucleotides due to the natural properties of the genome. Thus in order to streamline the oligonucleotide design for the following studies, we built a limited weight pool composed of uniqueness weights and GC content from 6 to 9, SW weight is 1 or 2, and LZ is defined as 1. This scheme creates new 32 optimized sets (combinations of w_BLAST_, w_GC_, w_SW_, w_LZ _= [6,7,8,9][6,7,8,9][1,2][1]) which can be used effective oligonucleotide selection. Based on these simulations, we expect that the 32 weight sets will provide sufficient flexibility and efficiency to optimize all four parameters, while preserving the higher importance of the GC content and uniqueness. In summary the OligoRankPick algorithm is capable of designing microarray oligonucleotide sets avoiding any arbitrary cutoff implemented directly in the selection algorithm. Instead this algorithm incorporates various empirical and theoretical boundaries via target values to optimize the simulation step during which specific weight intervals are determined.

### Comparison with other programs

**Figure 3:**
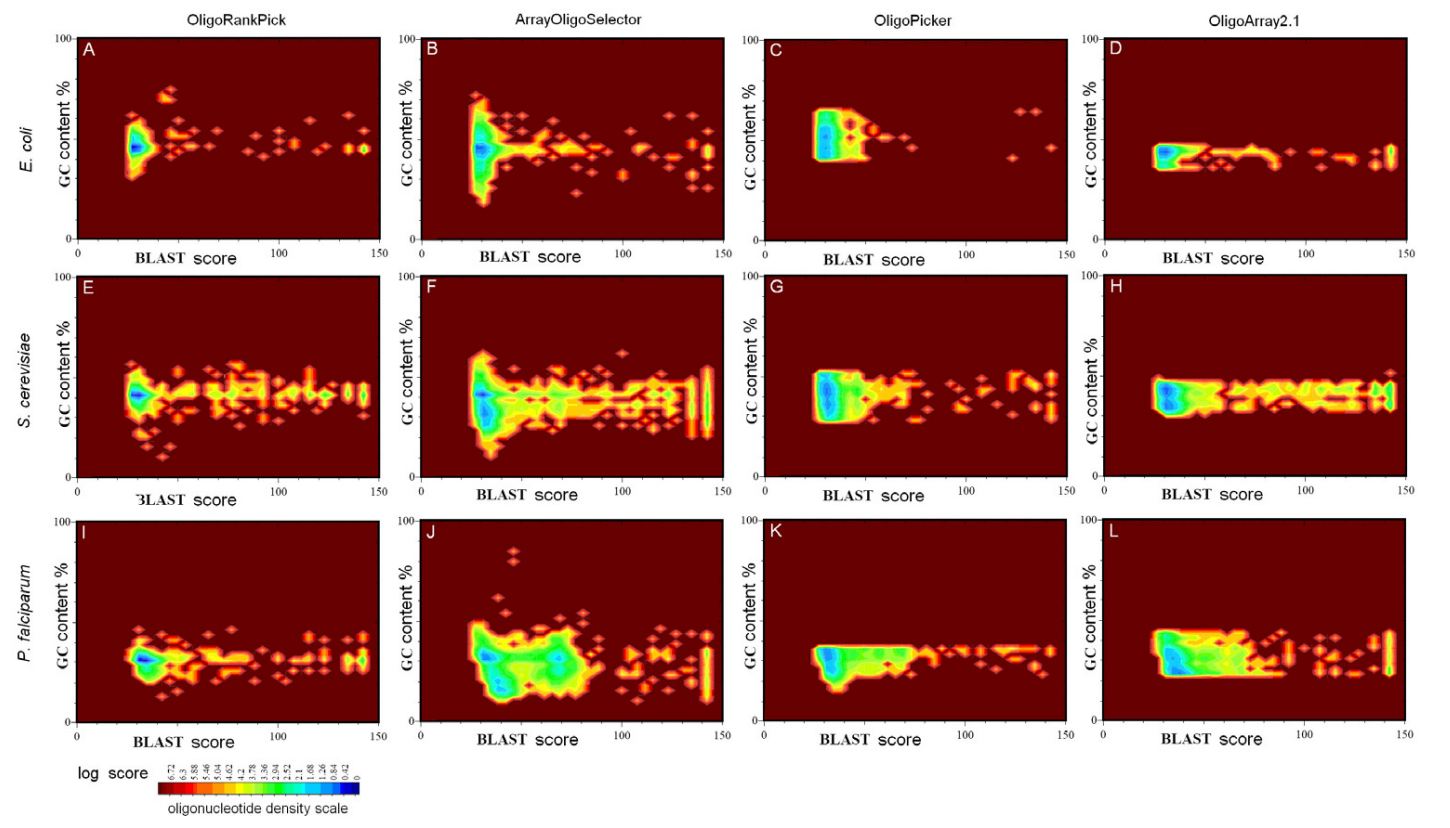
Overall profiles of the uniqueness and GC content of oligonucleotide microarray elements in the 12 designed theoretical microarray sets. Four algorithms OligoRankPick, ArrayoligoSelector, OligoPicker, and OligoArray2.1 were used to design long oligonucleotide DNA microarray sets for *P. falciparum*, *E. coli *and *S. cerevisiae*. Contour plots illustrate oligonucleotide density plotted of along the uniqueness scores (second target BLAST scores) and GC contents. The oligonucleotide density is is calculated as -log10(N/N_max_) (N ~ number of oligonucletide in a given area and N_max _~ number of oligonucleotide in the most dense area)and displayed using the indicated by the color based scale.

To compare the performance of OligoRankPick with other publicly available programs, we designed three theoretical microarray oligonucleotide sets for the *P. falciparum, S. cerevisiae *and *E. coli*. We selected three programs, ArrayOligoSelector [5], OligoPicker [8] and OligoArray 2.1 [7]. For the intended designs we chose the oligonucleotide length to be 70 nt and the GC content 31.4% (Tm = 74.7) for *P. falciparum*, 40% (Tm = 79.8) for *S. cerevisiae *and 45% for *E. coli *(Tm = 82.7). The theoretical oligonucleotide sets were designed using the publicly available sequence data and the selection algorithms with default settings. Figure 3 summarizes the parameter distributions of the uniqueness scores (BLAST scores of the final oligonucleotides to their second best genomic targets) plotted against GC content. Overall these contour plots illustrate that comparing to the three publicly available programs, OligoRankPick provides significant improvements for the design of yeast, *E. coli *and *P. falciparum *microarray (figure 3, see Additional file 2). The most striking improvements were, however, observed in the design of the *P. falciparum *microarray. For this genome the BLAST scores and the GC content of the oligonucleotides designed by OligoRankPick exhibit a greater convergence to a small region in the desired area (low BLAST scores, GC around 31.4%) compared to oligonucleotides designed by the three other programs (figure 3). Similar convergence is observed for the SW and LZ scores (see Additional file 1 figure S2 and S3). To further demonstrate the convergence of the oligonucleotide parameters we calculated a mean distance for each parameter distribution to its desired (preset) value and also to the average value within the parameter distribution (see Additional file 1 figure S4). In all cases the OligoRankPick produced the smallest mean distances and thus tighter distribution of the oligonucleotide parameters. The only exception is the lower mean distance of the CG content from its mean value in the yeast set designed by OligoPicker. Detailed inspection of these results indicated that the low mean distance is due to extensive filtering implemented by this program (data not shown). For each of the theoretical microarray dataset we also calculate the average weight score (AWS) which is directly related to the oligonucleotide quality with respect to the oligonucleotide parameters (for explanation see Additional file 1 figure S5). The smaller AWS that are consistently observed for the OligoRankPick generated oligonucleotide sets compared to the three other programs further indicate the optimization power of OligoRankPick.

Table 1 summarizes the overall statistics of the 12 oligonucleotide sets for different datasets and methods. Similar to weight score simulation we define the 40% continuous sequence identity to a second target and 5% deviation from the target GC content as the "good quality" criteria. OligoRankPick outperformed the other programs producing the highest number of oligonucleotides within the target limits (95.6%, 91.3% and 94.9% for *E. coli*, *S. cerevisiae *and *P. falciparum *respectively, table 1). The unbiased character of the OligoRankPick algorithm is also demonstrated by the total number of oligonucleotides designed. Since OligoRankPick does not use any filters, this method will select an oligonucleotide candidate for essentially any genetic locus (see "#designed" in table 1). There were only 5 coding sequences not considered by OligoRankPick in *E. coli *and one in *S. cerevisiae *due to their sequence lengths being shorter than 70 nt (table 1).

**Table 1:** The comparison of designed oligonucleotides from different programs

Programs*	*E. coli *K12 (4237 cds)		*S. cerevisiae *(6680 cds)		*P. falciparum *(5363 cds)	
	
	#designed^a^	#accepted ^b^	#designed	#accepted	#designed	#accepted
**OligoRankPick**	**4232^ξ^**	**4047(95.6)**^&^	**6679**^ζ^	**6096(91.3)**	**5363**	**5092(94.9)**
ArrayOligoSelector	4201	3371(80.2)	6221	3471(55.8)	5339	2093(39.2)
OligoPicker	4142	2594(62.6)	6208	3614(58.2)	4235	3543(83.7)
OligoAarray 2.1	3221	2826(87.7)	6587	4440(67.4)	5206	2317(44.5)

*ArrayOligoSelector 3.8.4; OligoPicker; OligoArray 2.1. a: oligonucleotide number selected by the program; b: good oligonucleotide number based on BLAST score of non-target (<= 40% continuous identity) and GC content (± 5%). & Percentage of good quality oligonucleotide to total selected oligonucleotide (in the bracket). ξ Five rejected coding sequences are less than 70 bp. ζ Only one rejected sequence is YJR151W-A (51 bp).

One of the unique features of the *P. falciparum *genome is the presence of several large highly homologous gene families whose role has been implicated in the antigenic variation including *var *(76 members), *rifin *(164 members) and *stevor *(34 members)[16,18]. Table 2 indicates the number of unique oligonucleotides designed by all the four programs for these genes. OligoRankPick was capable of designing unique oligonucleotides for 234 genes (85.4%) of total 274 genes which by far exceeded the performance of the three other algorithms.

### Design of a gene specific DNA microarray for *P. falciparum*

**Table 2:** The oligonulceotide design of large gene families from different programs

Programs*	*Var family *(Total No. 76)		*Rifin family *(Total No. 164)		*Stevor family *(Total No. 34)	
	
	#designed^a^	#accepted^b^	#designed	#accepted	#designed	#accepted
**OligoRankPick**	**76**	**63**	**164**	**140**	**34**	**31**
ArrayOligoSelector	76	31	162	58	34	13
OligoPicker	37	37	78	74	12	12
OligoAarray 2.1	22	9	162	118	34	22

*ArrayOligoSelector 3.8.4; OligoPicker; OligoArray 2.1. a: oligonucleotide number selected by the program; b: accepted oligonucleotide number based on BLAST score of non-target (< 40% continuous identity).

**Figure 4:**
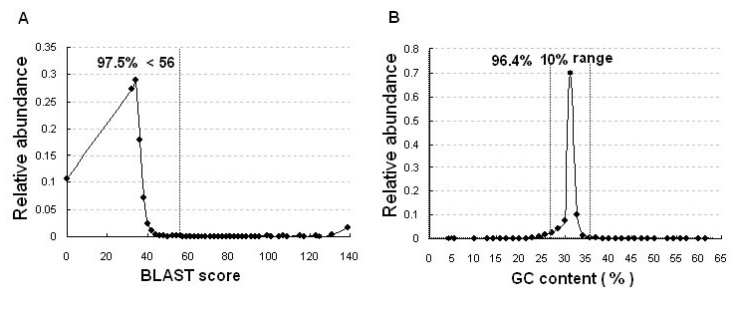
Oligonucleotide parameter distributions in the newly designed *P. falciparum *DNA microarray. Total 10166 oligonucleotides were designed for the P. falciprum DNA microarray. Relative abundance of the oligonucleotides is plotted along the uniqueness scores (BLAST score of the second-best target in the genome) **(A) **and along the GC content **(B)**. The dotted line indicates the quality control criteria (see text) with BLAST score = 56 which corresponding to > 40% continuous match cross-hybridization and the 31.4% ± 5% interval of GC content corresponding to the targeted range. Percentages of oligonucleotides which fall within the targeted values are indicated.

In the final step we applied OligoRankPick to design a gene specific DNA microarray for the *P. falciparum *genome (5363 coding sequences, CDS) that can be used for functional genomic studies of this important human pathogen. For this design we wished to increase the oligonucleotide coverage for longer open reading frames and thus we fragmented each coding sequence using the fragmentation.pl script as follows: sequences smaller than 1 kb were kept as one fragment; sequences between 1 kb and 2 kb were split evenly into two fragments, sequences larger then 2 kb were split into n fragments (n > = 2) when: (2n-2)kb < gene size > (2n)kb. The fragmentation step generated 10166 Microarray Element Fragments (MEFs) from 5363 CDS. A single oligonucleotide was designed for each MEF which resulted in one oligonucleotide per 1198 bp on average for all *P. falciparum *coding sequences. Although the median GC content of all 70 nt oligonucleotide windows in the *P. falciparum *coding sequences is 24.3% (displayed by GC_dis.pl optional module) for higher specificity and efficiency of microarray hybridization, we selected oligonucleotides with a GC content of 31.4% (22 GCs out of 70 nt). OligoRankPick successfully designed 10166 oligonucleotides representing all predicted *P. falciparum *genes with an average of 1.9 oligonucleotides per protein coding sequence (see Additional file 3). Figure 4B summarizes the GC content distribution suggesting that OligoRankPick can identify optimal oligonucleotide elements with GC content significantly distant from the average GC content in the genome. Astonishingly 70.5% of the designed oligonucleotides had the desired GC content of 31.4% (figure 4B).

To evaluate the level of uniqueness of the designed oligonucleotides we used the identical quality control criteria used for the weight optimization strategy which is consistent with previously established conditions of optimal microarray hybridization performance (see above). In total 9909 (97.5%) oligonucleotides passed the uniqueness criteria and 9795(96.4%) oligonucleotides were found to be in the range of 5% deviation from the GC content target value (31.4%) (figure 4). There are 9584 (94.7%) oligonucleotides meeting both criteria while only 275 oligonucleotides (2.7%) were outside of the ± 5% GC content interval and 257 oligonucleotides (2.5%) were not unique in the genome. Manual inspections of the MEFs represented by these oligonucleotides indicated that no suitable 70 nt window exists within these DNA fragments. The 257 non-unique oligonucleotides represented 193 genes (3.6% of total CDS) from which 67 genes belong to the large multigenic gene families, *var*,*rifin *and *stevor*. Pair-wise sequence homology analysis of these genes revealed that these genes do not contain any 70 nt window that shares less than 40% homology with any other member of the corresponding gene family and thus no unique oligonucleotide could be selected by any conceivable strategy. Interestingly for the remaining 185 (73.4%) members of these families a specific oligonucleotide was selected which further demonstrates the power of OligoRankPick for microarray design.

### Transcriptome analysis of the trophozoite and schizont stages of *P. falciparum*

Although all parameters of the oligonucleotide microarray sets designed by OligoRankPick indicate their high quality, the ultimate evidence for their functionality can be provided only by physical microarray experiments. For this purpose we have synthesized all the 10166 oligonucleotides for the *P. falciparum *genome-wide microarray and spotted these onto polylysine-coated microscopic slides as previously described [19]. Using these microarrays we compare the global mRNA patterns between two developmental stages of the *P. falciparum *intraerythrocytic development, trophozoite and schizont. All experimental procedures were carried out as previously described [5] and the complete results for three replicates of the microarray hybridizations are available in the supplementary data. The *P. falciparum *genome sequence reference strain 3D7 was used for this analysis. Total 4183 genes were found to be expressed in at least one of the studied developmental stages in three replicates of microarray hybridization. From these 1891 and 841 mRNA transcripts exhibited at least 2-fold higher abundance in the trophozoite and the schizont stage, respectively (see Additional file 4).

In order to assess the fidelity of the obtained results we wish to compare this data to previously published transcriptome analyses of the *P. falciparum *intraerythrocytic developmental cycle (IDC). These include the IDC transcriptome analyzed by the previous version of a long oligonulceotide microarray (LOM-IDC transcriptome) comprised of 2689 genes [20], and a high density short oligonucleotide Affymetrix microarray dataset (HDSO-Affymetrix transcriptome) comprised of 1162 genes with stage specific transcription [21]. All genes present in both LOM-IDC and HDSO-Affymetrix transcriptomes were represented on the new *P. falciparum *microarray and yielded a hybridization signal in at least two of the three microarray replicates. To compare the stage specificity of the gene expression we select genes which exhibited > 3-fold change in mRNA abundance between trophozoite and schizonts detected in at least two (out of three) replicates (table 3). Using these criteria we classify 862 genes as trophozoite specific and 431 genes as schizont specific. The transcriptome data comparisons, summarized in table 3, indicate high correlations between the transcriptome data and the new microarray dataset with 91.2–95.9% of overlapping genes exhibiting identical stage specificity in their mRNA levels. There were only a small number of genes (8.8–4.1%) for which the new expression results did not correlate with the previously published data. These discrepancies are likely caused by subtle differences in parasite culture synchronicity and stage representation between our culturing system and the systems used for the previous transcriptome analyses.

**Table 3:** *P. falciparum *microarray data and their comparisons to existing transcriptomes

Transcriptome results	**Trophozoite**	**Schizont**
**3-fold in at least two replicates**	**862**	**431**

Present in the LOM-IDC transcriptome	630/73%	320/74.2%
*Same stage classification in LOM-IDC Transcriptome	595/94.5%	307/95.9%
Present in the HDSO-Affymetrix transcriptome	741/86%	353/82%
**Same stage classification in HDSO-Affymetrix transcriptome	676/91.2%	336/95.2%

*genes with peak expression before and after 30 hours post invasion are classified as trophozoite and schizont specific, respectively

** genes with higher expression levels in late ring and early and late trophozoites compared to early and late schizonts are classified as trophozoite specific and *vice versa*

**Figure 5:**
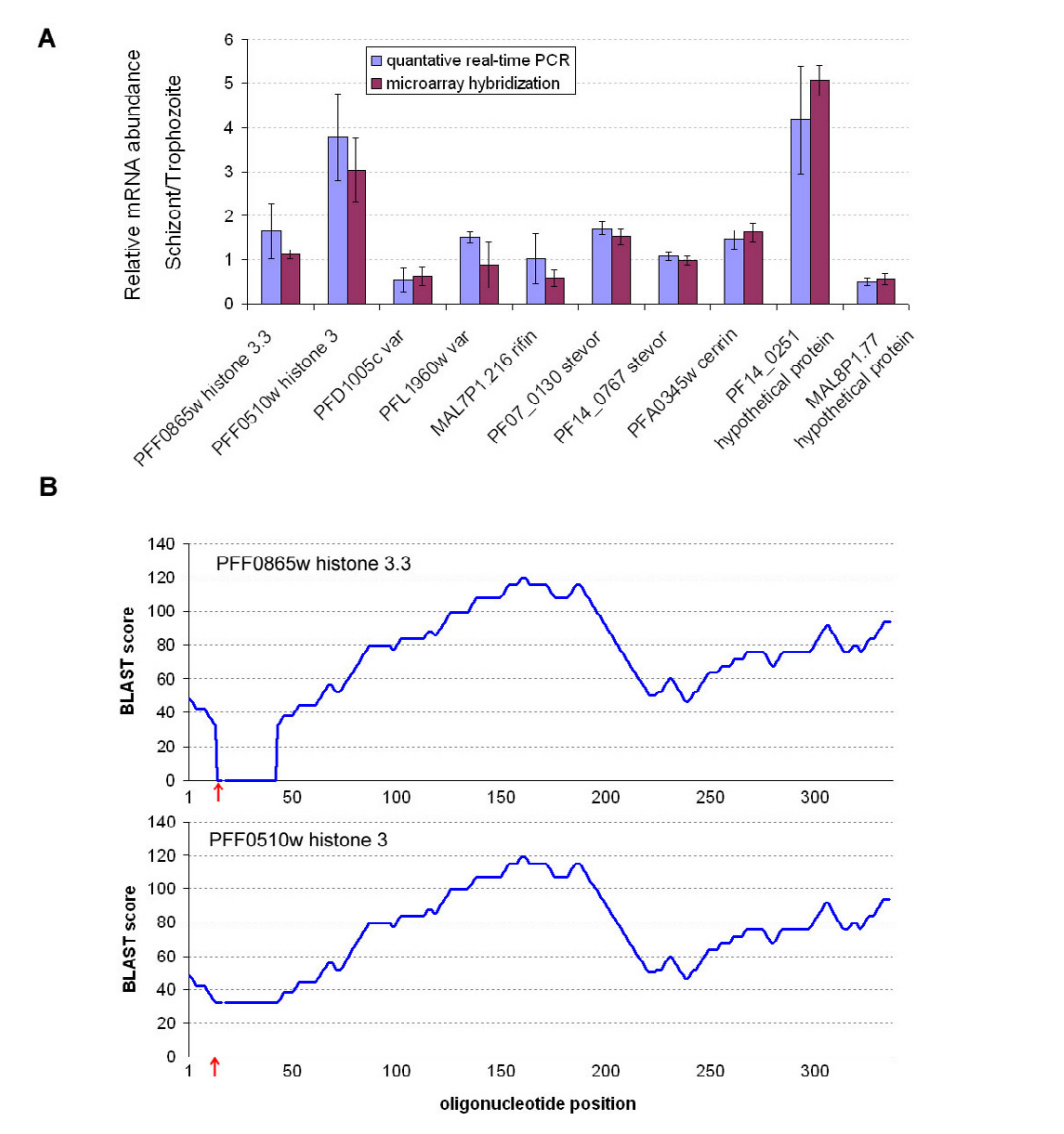
Verifications of microarray results by quantitative real-time PCR **(A) **and example of oligonucleotide selection for highly homologous genes **(B)**. The bar graph indicates mRNA abundance ratios between two developmental stages (schizont/trophozoite) of the *P. falciparum *IDC for 10 genes measured by microarray and by real-time RT-PCR. The expression data were obtained using the total RNA isolations from the trophozoite and schizont stage. Each measurement was carried three times and the standard error for each measurement is indicated. **(A)**. The uniqueness score distributions along the two highly homologous histone 3 genes. The uniqueness is represented by the BLAST score of each 70 nt window along the histone genes (H3 and H3.3) to its second best target in the genome. The red arrow indicates the position of oligonucleotide selected by OligoRankPick in each gene **(B)**.

To further validate the performance of the designed *P. falciparum *microarray quantitative real-time RT-PCR was used to measure relative mRNA abundance between trophozoite and schizont stage for 10 selected genes. For this we chose genes for which only OligoRankPick designed a "good quality" microarray element while the three tested publicly available programs did not yield a suitable oligonucleotide element. These include two paralogous histone3, five members of the variable surface antigen gene families (2 *var*, 1 *rifin*, 2 *stevor*), centrin, and two genes encoding highly homologous hypothetical proteins. Figure 5A shows good correlations between the RT-PCR results and microarray hybridization data which demonstrate the robust performance of the newly designed microarray for analyses of mRNA abundance in *P. falciparum*. Detail sequence analyses revealed that each of the 10 selected genes contains only a small window of unique sequence while the majority of the gene is highly homologous to at least one other locus in the genome. One of the example is a pair of highly homologous genes encoding histone3 (H3) and its homologue histone3.3 (H3.3) (figure 5B). This high homology is likely the main obstacle for designing a specific oligonucleotide and it is the reason why no transcription data have been obtained by the previously reported transcriptome analyses. Despite this OligoRankPick selected specific oligonucleotides which overlap the most unique region of each gene (figure 5B). The microarray hybridization signal detected on these oligonucleotide elements revealed that these two highly homologous genes undergo different transcription regulation during the IDC with H3 exhibiting 3-fold increase of mRNA abundance in schizonts compare to trophozoites and H3.3 showing similar amounts (< 2-fold change) between these two developmental stages (figure 5A).

Taken together these data demonstrate that the newly designed microarray for *P. falciparum *successfully recapitulates data from previous transcriptome analyses and has a potential to further expand on these results. Overall these data verify the improved performance of OligoRankPick in designing unique microarray elements for gene expression microarrays

## Discussion

The main goal of this work was to develop a microarray design algorithm which combines the thoroughness of the parameter optimization methods (such as CommOligo [15]) and performs with high computational efficiency of the earlier, cutoff based techniques (such as OligoArraySelector [5]). The newly developed algorithm, OligoRankPick, is the first method using a parameter optimization approach that is computationally fast and robust for genome-wide microarray design. The core principle of this technique consists of the rank transformations of the parameter scores and the subsequent weighted rank-sum strategy. This allowed us to eliminate all cutoff based filters that are typically applied to the input data (by existing optimization programs) or to partial oligonucleotide lists that are generated prior or during the decision-making step (in cutoff-based methods). Instead the derived rank-based system maintains all the oligonucleotide candidates in their rank order throughout the entire process. This approach removes any ambiguities in the selection process as all oligonucleotides are constantly prioritized based on their properties. Since no oligonucleotides are eliminated by arbitrary cutoffs, this method also significantly expands the genome coverage of the designed microarrays. The simplicity of the rank-based approach also allows the algorithm to perform gene specific optimizations of the weight coefficients in which the contribution of each parameter is modified based on the sequence properties of a particular gene. This is especially useful for optimal probe design in genes with extreme parameters distributions such as high AT content or high sequence homology to other genomic locus (low uniqueness). For example AT richness of some genes causes the GC content parameter to be over emphasized due to a stronger priority that is given to the GC rich oligonucleotide windows. This could force a selection of less unique oligonucleotides or oligonucleotides with complex secondary structure from these CG rich oligonucleotide candidates. The implementation of the gene specific optimizations is likely the most innovative approach introduced by this method because it generates a tighter distribution for each oligonucleotide parameter compared to other publicly available programs (figure 3). For general functionality we derived and validate optimal weight set intervals which could be applied to a wide range of genomes. The flexibility of the OligoRankPick package, however, allows the users to tune these setting for other specialized applications.

For the development and validation of OligoRankPick we design a new DNA microarray for the most lethal species of the human malaria parasites *P. falciparum *whose genome was completed in 2002 [16]. We chose this genome for its extreme AT/GC distribution and high level of gene duplication do demonstrate the utility of the newly design program for its future applications. The average GC content in the *P. falciparum *genome is estimated 19.4% (23.7% in coding and 13.5% in non-coding sequences). For this design, however, we wished to select oligonucleotides with higher GC content to ensure higher Tm and thus specificity and selectivity of each probe. In addition the requirement for high GC content will help to select oligonucleotides with high sequence complexity as AT rich sequences in *P. falciparum *contain numerous short nucleotide repeats. As demonstrated in figure 3 OligoRankPick was able to design a set of oligonucleotides whose GC content is tightly distributed around 31.4%. At the same time high levels of uniqueness and sequence complexity and a low level of secondary structures were preserved in the vast majority of the probes. This feature of OligoRankPick will be particularly useful for microarray design of many organisms with extreme fluctuations in GC content such as *Mycoplasma mycoides *[22] and other bacterial species [23], other "AT rich" *Plasmodium spp*. [24] and *Dictyostelium discoideum *[25] or GC rich *Leishmania spp*. [26]. The *P. falciparum *genome was found to contain a large number of duplicated genes sharing high levels of homology [16]. The extreme examples are the three gene families (*var, rifin, stevor*) which are involved in the parasite virulence and are presently explored as potential molecular targets for malaria intervention strategies [27]. Despite the high levels of homology amongst the individual members of these gene families, OligoRankPick was capable of designing specific oligonucleotide representative for 74.3% of these genes which by far exceeded the performance of the three tested publicly available programs. This improved performance will render OligoRankPick useful for studies of many organisms with highly homologous, biologically significant gene families ranging from microbial pathogens [28] to high eukaryotes [29].

## Conclusion

OligoRankPick provides a powerful alternative for long oligonucleotide microarray design for genomes with extreme GC content fluctuations and high abundance of highly homologous gene families. In its simplest implementation a user needs only to define the probe length and an expected GC content or Tm. However, for specialized applications, OligoRankPick provides the user with the option of setting the range of relative importance (weight) of each parameter as well as optimization of the quality control target values. Using this method we have designed and assembled a next generation of long oligonucleotide DNA microarray for the main parasitic species of human malaria *P. falciparum*. Transcriptome analyses of two *P. falciparum *developmental stages demonstrated that the designed microarray provides the most comprehensive coverage of the *P. falciparum *genome presently available.

The oligonucleotide sequences and the transcriptome data are available from the supplementary file.

## Methods

### Genome sequences and annotations

The *E. coli *gene sequence file with 4237 CDSs and genomic sequence file were downloaded from the NCBI genome database. The *S. cerevisiae *gene sequence file with 6680 CDSs, and whole genome sequence file were downloaded from the ENSEMBL database[30]. The *P. falciparum *protein coding sequence file with 5363 coding sequences (CDSs) and whole genomic sequence file were downloaded from PlasmoDB version 4.4 [31].

### The OligoRankPick Program

#### Implementation

The OligoRankPick is divided into two parts (two scripts). The first script (oligoblast.pl) is used to generate all possible oligonucleotides and their parsed BLAST results including its first, second and third best hybridization target (top three). The oligoblast.pl script can be run on different computers or a computer cluster using parallel processing methods such as mpiBLAST [32] and the results should be parsed according to the format of oligoblast.pl output. The second script (oligorankpick.pl) selects the optimal oligonucleotide for each sequence. There are four additional scripts which can be used to optimize the OligoRankPick package performance including masker.pl, used to mask the repeat sequence based on the NCBI dust program; GC_dis.pl, used to plot the GC content distribution of all oligonucleotides in the dataset in order to define a suitable GC content; fragmentation.pl, used to partition the long sequences to increase the oligonucleotide density in the coding sequences (see *P. falciparum *microarray design); simulation_ws.pl, used to modify the weight set file (wt_pool.opt) for special genomes.

#### Parameters of oligonucleotide measurements

For each input sequence OligoRankPick uses a sliding window of a given size (user setting, e.g. 70 nucleotides) to produce all possible oligonucleotides and calculates four parameters for each oligonucleotide: uniqueness (NCBI-BLAST score to its second best genomic target), GC content, secondary structure (reverse Smith-Waterman score, SW), and sequence complexity (LZ) (figure 1). (i) OligoRankPick uses the bit score of the second best match within the genome to calculate the level of oligonucleotide specificity using the NCBI-BLAST program version 2.0 [33]. The input values for the BLAST algorithm are adjusted as follows, -e 1 (E-value < 1) and -b 20 (maximum output items = 20) to limit the computer-time consumption; -m 8 (tabular output is chosen for more efficient parsing). (ii) To ensure the uniformity of the hybridization temperature of the microarray elements, strict criteria for GC content are implemented. Perl script (GC_dis.pl) is provided to evaluate the GC distributions for all possible oligonucleotides in the input. Users can convert melting temperature (Tm) into GC content using the following formula [34]: GC content = (Tm – 64.9)/41 + 600/(41*oligo length) [34]. OligoRankPick calculates the absolute deviation of the oligonucleotide GC content (Tm) from the desired value for the final oligonucleotide selection. (iii) OligoRankPick uses the reverse Smith-Waterman algorithm with the PAM47 DNA matrix to calculate the optimal alignment score between the candidate oligonucleotide sequence and its reverse complement sequence [35] to avoid any complex secondary structures which can be detrimental to hybridization performance. (iv) OligoRankPick uses the compression score which is calculated by the Lempel-Ziv algorithm (LZ score), to avoid the presence of low-complexity sequences. These typically signal a presence of short nucleotide repeats that could result in significant non-specific cross-hybridizations. The use of the Lempel-Ziv (LZ) compression algorithm [36] was first introduced by Wright and Church [6] and further explored by ArrayOligoSelector [5]. This approach was particularly useful in elimination of short repetitive sequences during the oligonucleotide design for the AT-rich *P. falciparum *genome that contains a large number continuous stretches of A and T nucleotides in both the non-coding and the coding regions.

#### Selection of optimal oligonucleotides by Rank-sum strategy

In the next step OligoRankPick ranks all possible oligonucleotides in one locus according to their parameter scores and assigns an ordinal number for each parameter. While the BLAST, SW, LZ score are directly transformed into a rank, the GC content scores are first transformed to their absolute deviation from the defined GC content. Oligonucleotides with an identical score for any parameter are offered the same rank number. Subsequently the rank-sum strategy is used to select the optimal oligonucleotide(s). This strategy is based on the calculation of a weight rank-sum of all four ranks for each oligonucleotide within a locus by a linear function utilizing the following formula (also see figure 1):

RSk=Mink(∑j=14wj∗Rjk)     (1)
 MathType@MTEF@5@5@+=feaafiart1ev1aaatCvAUfKttLearuWrP9MDH5MBPbIqV92AaeXatLxBI9gBaebbnrfifHhDYfgasaacH8akY=wiFfYdH8Gipec8Eeeu0xXdbba9frFj0=OqFfea0dXdd9vqai=hGuQ8kuc9pgc9s8qqaq=dirpe0xb9q8qiLsFr0=vr0=vr0dc8meaabaqaciaacaGaaeqabaqabeGadaaakeaacqqGsbGucqqGtbWudaWgaaWcbaGaee4AaSgabeaakiabg2da9maaxababaGaeeyta0KaeeyAaKMaeeOBa4galeaacqqGRbWAaeqaaOGaeiikaGYaaabmaeaacqqG3bWDdaWgaaWcbaGaeeOAaOgabeaakiabgEHiQiabbkfasnaaBaaaleaacqqGQbGAcqqGRbWAaeqaaOGaeiykaKcaleaacqWGQbGAcqGH9aqpcqaIXaqmaeaacqaI0aana0GaeyyeIuoaaaa@4725@

Where W_j _is the weight of the j-th parameter (j = 1, 2, 3, 4), R_jk _is rank score of j-th parameter of the k-th oligonucleotide (k = 1, ..., n). In the first step the rank-sum function selects the oligonucleotide with the minimal rank-sum (RS) as the candidate for one given weight set.

The weight coefficients reflect the relative importance of each particular parameter for the final selection. In our implementation the initial levels of the each parameter importance was derived from previous empirical experience with spotted long oligonucleotide microarray technology [5,37,38]. To accommodate the variable characteristics of the DNA sequence along the genome we introduce an additional step in which the optimal weight values are determined for each gene individually. There is a weight file (wt_pool.opt) to offer the optimal intervals of weight values for the user from the simulation for "standard" microbial genomes (showed in the following section). However, users can define specific weights and modify this file based on their own theoretical or empirical experience as well as specific requirements (simulation_ws.pl provided in the package). For all sets of weights:

TO=Mini(RSKi/∑wi)     (2)
 MathType@MTEF@5@5@+=feaafiart1ev1aaatCvAUfKttLearuWrP9MDH5MBPbIqV92AaeXatLxBI9gBaebbnrfifHhDYfgasaacH8akY=wiFfYdH8Gipec8Eeeu0xXdbba9frFj0=OqFfea0dXdd9vqai=hGuQ8kuc9pgc9s8qqaq=dirpe0xb9q8qiLsFr0=vr0=vr0dc8meaabaqaciaacaGaaeqabaqabeGadaaakeaacqqGubavcqqGpbWtcqGH9aqpdaWfqaqaaiabb2eanjabbMgaPjabb6gaUbWcbaGaeeyAaKgabeaakiabcIcaOiabbkfasjabbofatnaaBaaaleaacqqGlbWscqqGPbqAaeqaaOGaei4la8YaaabqaeaacqqG3bWDdaWgaaWcbaGaeeyAaKgabeaaaeqabeqdcqGHris5aOGaeiykaKcaaa@4228@

Where RS_Ki _is the optimal selected oligonucleotide (K oligonucleotide) for weight set i, ∑w_i_ is the sum of weights for weight set i. TO (Target Oligonucleotide) is the final selected oligonucleotide. The optimization step (formula 2) is performed for all weight sets reflecting all combination of weight values in the input intervals. Oligonucleotides with the minimal RS from each weight set (RS_Ki_) are transformed ("normalized") by the sum of weight values for the four parameters. The oligonucleotide with the minimum RS value is the optimal local solution of the rank-sum function in the given weight set interval (figure 1). This oligonucleotide is chosen as the final candidate.

#### Microarray hybridization and quantitative real-time PCR

Microarray hybridizations were conducted as previously described [5]. Real time RT PCR was performed in a total reaction volume of 20 μl which contained 1 μl cDNA template (10 ng/ul), 0.5 μl forward and reverse primer (10μM), and 10 μl of 2 × Power SYBR Green PCR Master Mix (Applied Biosystems). The temple cDNA was generated using the first strand cDNA synthesis protocol used for the microarray hybridization. For the amplification the universal thermal cycling parameters were programmed as follows: 5 min activation at 95°C, followed by 40 cycles of 20s at 95°C, 30s at 50°C, 40s at 72°C and 1 min at 60°C. Each reaction was run in triplicates. The mRNA abundance ratios were calculated using ABI 7500 Fast Real-Time PCR Systems and the relative quantitation of gene expression was performed using the comparative CT method. Primers for PCR were designed using DNAMAN (Lynnon Corporation).

## Availability and requirements

Project name: OligoRankPick;

Project home page: ;

Operating system: Linux;

Programming language: Perl, C;

Licence: GNU GPL.

## Competing Interests 

The author(s) declares that there are no competing interests.

## Authors' contributions

GAH and ZB developed the program, conducted the majority of data analysis, all the transcription experiments and drafted the paper. ML, JL and PRP provided substantive contribution to the data analyses and helped to finalize the manuscript. All authors read and approved the final manuscript.

## Supplementary Material

Additional file 1 supplementary figure 1 to 5. Five supplementary figures supporting the presented results. Click here for file

Additional file 2 All oligonucleotides in table 1. 3 sets of the theoretical oligonucleotide microarrays for *E. coli *K12, *S. cerevisiae *and *P. falciparum*. Click here for file

Additional file 3 All gene-specific oligonucleotides for *P. falciparum*. The full set of oligonucleotides designed for the *P. falciparu *microarray. Click here for file

Additional file 4 Trophozoite and schizont transcriptome of *P. falciparum*. Genome-wide expression data for the trophozoite and schizont stages of the *P. falciparum *IDC. Click here for file
