# Supplementary material for: Selection of long oligonucleotides for gene expression microarrays using weighted rank-sum strategy
Source: BMC Bioinformatics. 2007 Sep 19;8:350. doi: 10.1186/1471-2105-8-350 (PMC2099447; doi:10.1186/1471-2105-8-350)
Supplement: Additional file 1 — supplementary figure 1 to 5. Five supplementary figures supporting the presented results. [file 1471-2105-8-350-S1.pdf]

Supplementary Figures

Figure S1 Correlations between the oligonucleotide parameter scores and their rank transformations along genes. Spearman rank order correlation coefficient were calculated between the parameter and rank-transformed profiles for the 5363 *P. falciparum* coding sequences. The calculated coefficients were plotted in ascending order for all four parameters. In no case a Spearman coefficient dropped below 0.98 with vast majority reaching the maximum value 1. This indicates that the rank transformation does not affect the oligonucleotide status in the oligonucleotide set. (Null hypothesis test, P˜ 0).

Figure S2 The SW (self-binding) score and GC content distributions for the designed oligonucleotide sets. In the each scatter plots SW scores (X-axis) is plotted against the GC content (Y-axis) for all oligonucleotides in the set. Total 12 oligonucleotide sets were designed for three genomes *E. coli* (A-D), *S. cerevisiae* (E-H), and *P. falciparum* (I-L) using all programs OligoRankPick (A, E, I), ArrayOligoSelector (B, F, J), OligoPicker(C, G, K), and OligoArray 2.1 (D, H, L). Tighter distribution the SW scores indicates the improved performance of OligoRankPick for microarray design.

Figure S3 The LZ (sequence complexity) score and GC content distributions for the designed oligonucleotide sets. In the each scatter plots the LZ scores (X-axis) are plotted against the GC content (Y-axis) for all oligonucleotides in the set. Total 12 oligonucleotide sets were designed for three genomes *E. coli* (A-D), *S. cerevisiae* (E-H), and *P. falciparum* (I-L) using all programs OligoRankPick (A, E, I), ArrayOligoSelector (B, F, J), OligoPicker(C, G, K), and OligoArray 2.1 (D, H, L). Tighter distribution the LZ scores indicates the improved performance of OligoRankPick for microarray design.

Figure S4 Analyses of the uniqueness and GC content distributions in the 12 designed theoretical microarray sets. A, single-parameter mean distances of BLAST score and GC content were calculated from all oligonucleotide scores to their mean score, respectively; B, the mean distances of BLAST score and GC content calculated from all oligonucleotide scores to the expected score. The expected BLAST score is the smallest one in all sets and the expected GC content is the defined GC content in the program; C and D shows the two-dimensional mean distances BLAST score and GC content calculated from all oligonucleotide points to their central point. The central point in C comprises the mean BLAST score and mean GC content in the oligonucleotide set. The central point in D has the smallest BLAST score and defined GC content.

Figure S5 The distribution of Rank status and Average weight score of the selected oligonucleotides from three datasets by different programs.A-D, the oligonucleotide sets of *E.coli* by four programs; E-H, the oligonucleotide sets of *S.cerevisiae*; I-L, the oligonucleotide sets of

P .falciparum. In each diagram, the top-right small diagram is the distribution of AWS (average weight score) of the whole oligonucleotide set, and the diagram below is the rank distribution of the whole oligonucleotide set. For AWS and rank status, the weight set I was first determined by ORP, then all oligonucleotide AWSs were calculated by formula 2 and ranked. The AWS and rank status of all selected oligonucleotides were mapped.

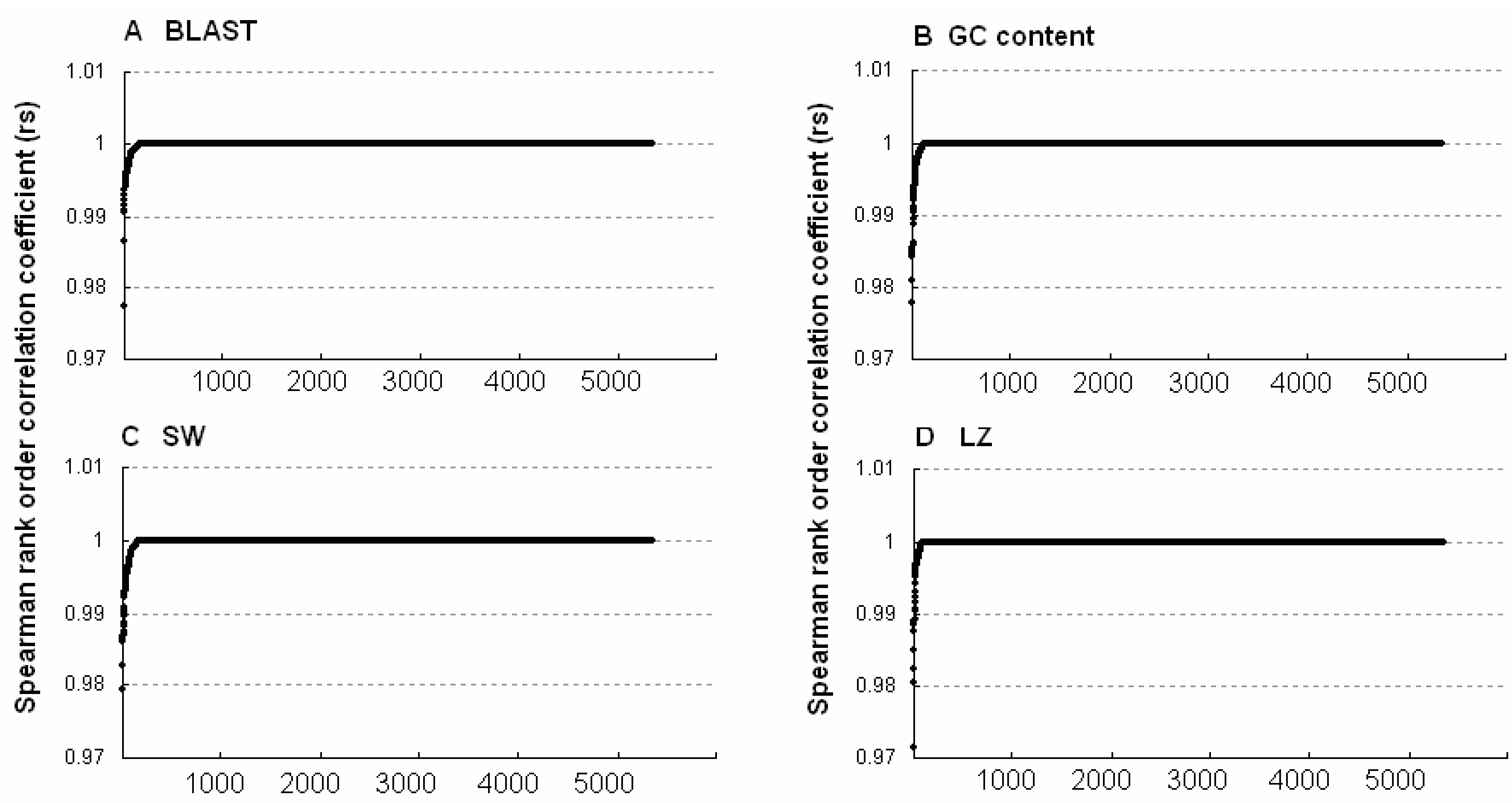
A BLAST
B GC content
C SW
D LZ
Spearman rank order correlation coefficient (rs)
1.01
1
0.99
0.98
0.97
1000
2000
3000
4000
5000

Figure S1

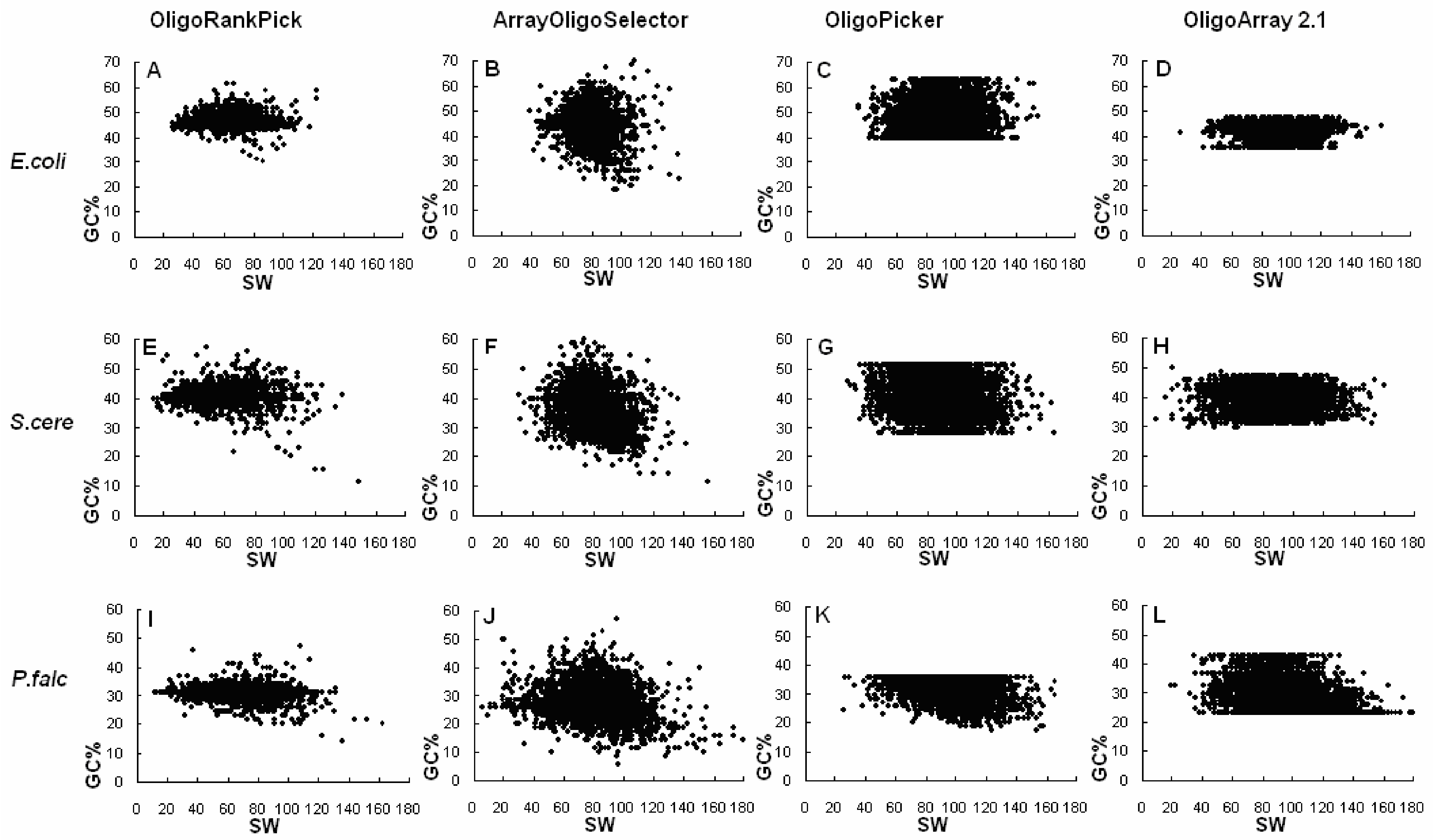
OligoRankPick
ArrayOligoSelector
OligoPicker
OligoArray 2.1
E.coli
S.cere
P.falc
A
B
C
D
E
F
G
H
I
J
K
L
GC%
SW

Figure S2

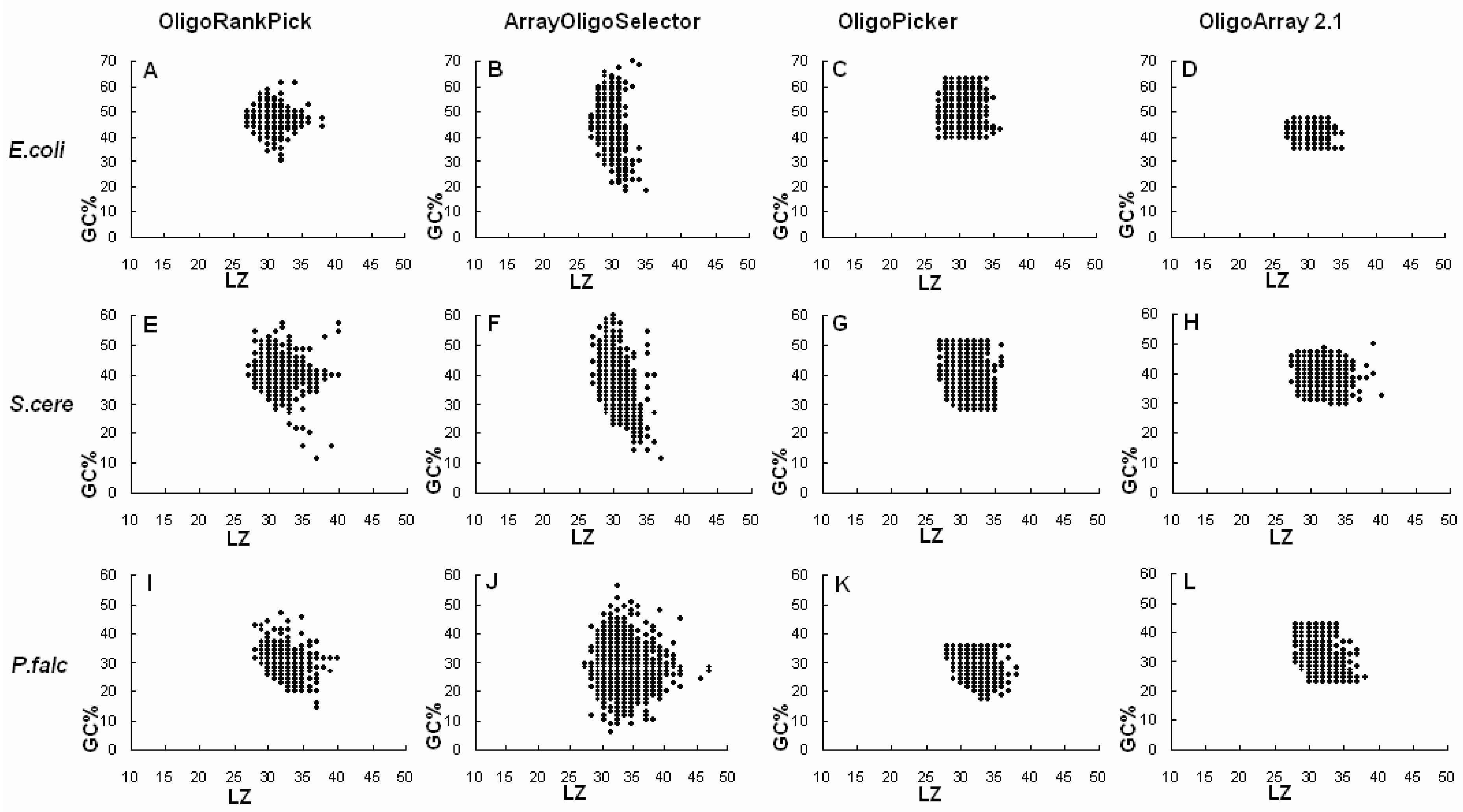
OligoRankPick
ArrayOligoSelector
OligoPicker
OligoArray 2.1
E.coli
S.cere
P.falc
A
B
C
D
E
F
G
H
I
J
K
L
GC%
LZ

Figure S3

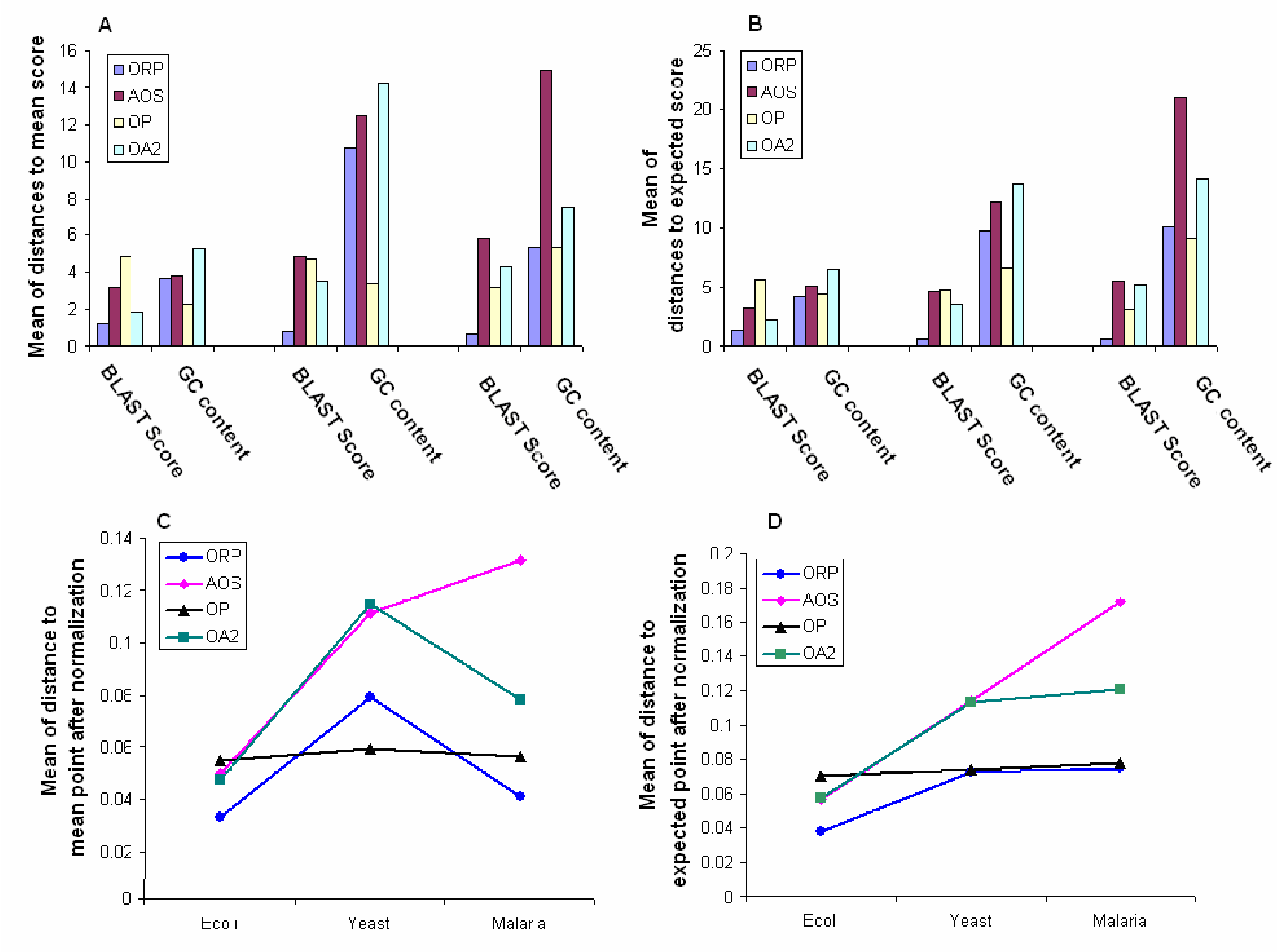
A
Mean of distances to mean score
16
14
12
10
8
6
4
2
0
ORP
AOS
OP
OA2
BLAST Score
GC content
BLAST Score
GC content
BLAST Score
GC content
B
Mean of
distances to expected score
25
20
15
10
5
0
ORP
AOS
OP
OA2
BLAST Score
GC content
BLAST Score
GC content
BLAST Score
GC content
C
Mean of distance to
mean point after normalization
0.14
0.12
0.1
0.08
0.06
0.04
0.02
0
ORP
AOS
OP
OA2
Ecoli
Yeast
Malaria
D
Mean of distance to
expected point after normalization
0.2
0.18
0.16
0.14
0.12
0.1
0.08
0.06
0.04
0.02
0
ORP
AOS
OP
OA2
Ecoli
Yeast
Malaria

Figure S4

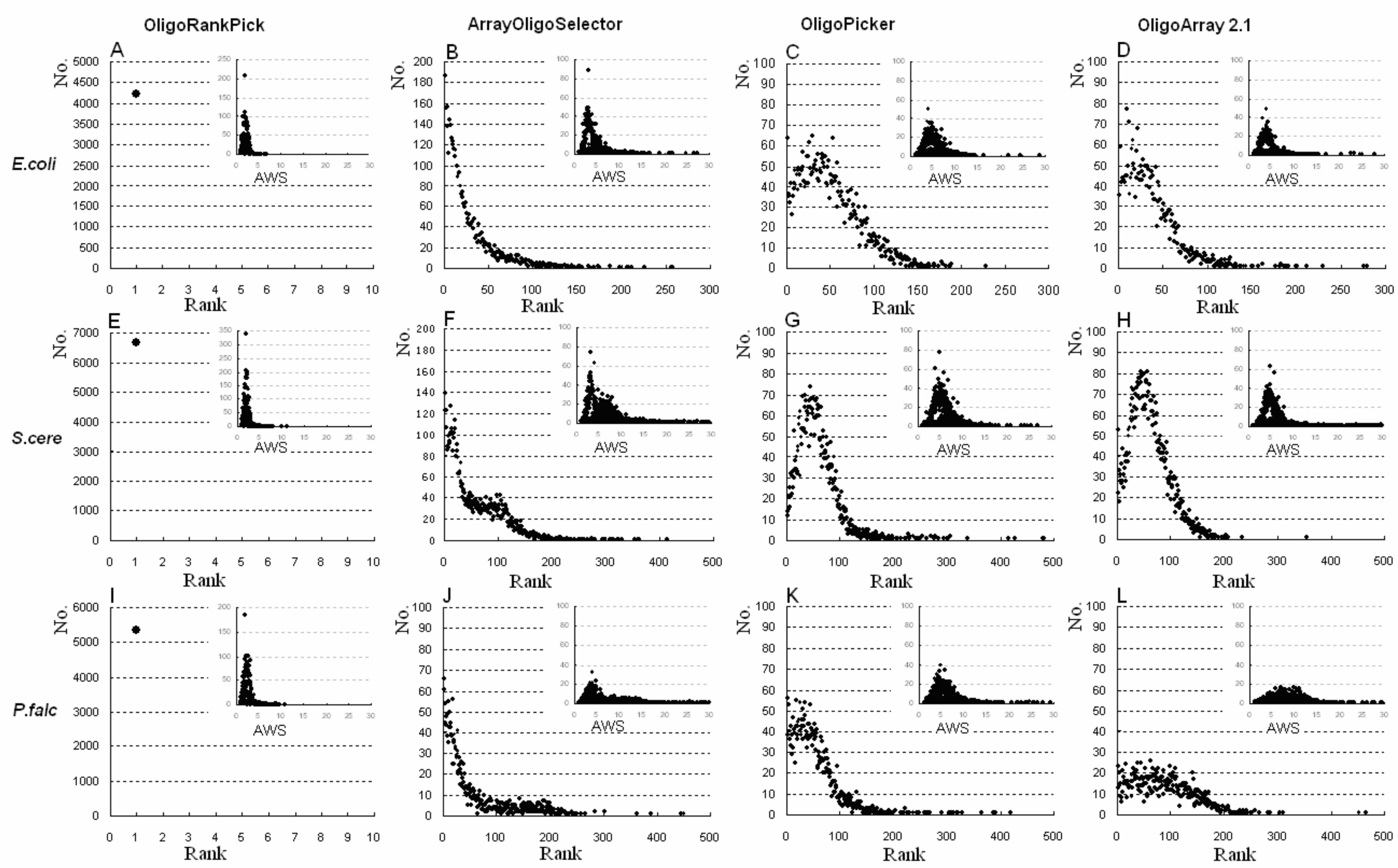
OligoRankPick
ArrayOligoSelector
OligoPicker
OligoArray 2.1
E.coli
S.cere
P.falc
A
B
C
D
E
F
G
H
I
J
K
L
No.
Rank
AWS

Figure S5
